# Bone Marrow-Derived Cells from Male Donors Do Not Contribute to the Endometrial Side Population of the Recipient

**DOI:** 10.1371/journal.pone.0030260

**Published:** 2012-01-19

**Authors:** Irene Cervelló, Claudia Gil-Sanchis, Aymara Mas, Amparo Faus, Jaime Sanz, Federico Moscardó, Gema Higueras, Miguel Angel Sanz, Antonio Pellicer, Carlos Simón

**Affiliations:** 1 Fundación IVI-Instituto Universitario IVI-Universidad de Valencia, INCLIVA, Valencia, Spain; 2 Servicio de Hematología, Hospital Universitario La Fe, Valencia, Spain; 3 Servicio de Ginecología, Hospital Universitario La Fe, Valencia, Spain; 4 Stem Cell Bank, Centro de Investigación Príncipe Felipe, Valencia, Spain; University of Medicine and Dentistry of New Jersey, United States of America

## Abstract

Accumulated evidence demonstrates the existence of bone marrow-derived cells origin in the endometria of women undergoing bone marrow transplantation (BMT). In these reports, cells of a bone marrow (BM) origin are able to differentiate into endometrial cells, although their contribution to endometrial regeneration is not yet clear. We have previously demonstrated the functional relevance of side population (SP) cells as the endogenous source of somatic stem cells (SSC) in the human endometrium. The present work aims to understand the presence and contribution of bone marrow-derived cells to the endometrium and the endometrial SP population of women who received BMT from male donors. Five female recipients with spontaneous or induced menstruations were selected and their endometrium was examined for the contribution of XY donor-derived cells using fluorescent *in situ* hybridization (FISH), telomapping and SP method investigation. We confirm the presence of XY donor-derived cells in the recipient endometrium ranging from 1.7% to 2.62%. We also identify 0.45–0.85% of the donor-derived cells in the epithelial compartment displaying CD9 marker, and 1.0–1.83% of the Vimentin-positive XY donor-derived cells in the stromal compartment. Although the percentage of endometrial SP cells decreased, possibly being due to chemotherapy applied to these patients, they were not formed by XY donor-derived cells, donor BM cells were not associated with the stem cell (SC) niches assessed by telomapping technique, and engraftment percentages were very low with no correlation between time from transplant and engraftment efficiency, suggesting random terminal differentiation. In conclusion, XY donor-derived cells of a BM origin may be considered a limited exogenous source of transdifferentiated endometrial cells rather than a cyclic source of BM donor-derived stem cells.

## Introduction

In the absence of pregnancy, the endometrial lining is removed every month and a new one is rebuilt under the control of steroid hormonal signals. This regenerative process occurs incessantly some 400 times throughout women's reproductive lifespan [1].

It has been associated with the existence of a relevant resident somatic stem cell (SSC) population in this tissue. Different groups have suggested the existence of this endometrial SSC in mice [2]–[3] and in humans [4]–[17], and to be hypothetically located near vessels in the basalis [18]–[21].

The side population (SP) phenotype, defined as cells capacity to extrude the DNA binding dye (Hoechst 33342) via the ATP-binding cassette transporter, was originally described as a marker of bone marrow stem cells (SC) [22]. It has been used to identify SC from many organs and tissues, such as the mammary gland [23], skin [24], myometrium [25], lung [26] and dental pulp [27].

In the human endometrium, several independent groups have demonstrated that SP cells functionally contribute to *in vitro* and *in vivo* tissue regeneration [18], [20], [28]–[29].

Bone marrow (BM) cells are able to differentiate into multiple nonhematopoietic cells [30]–[31]. In fact in human recipients who have received bone marrow transplantation (BMT), donor-derived cells have been proven to differentiate into hepatocytes [32], neurons [33], cardiomyocytes [34] and skin [35]–[36].

Donor-derived cells that are histologically and immunohistochemically similar to epithelial and stromal cells have been detected in the endometria of BM recipients [37]–[40]. This finding suggests a potential exogenous source for endometrial regeneration that can take place after BM mobilization as a normal physiologic process or under pathological conditions; e.g., chemotherapy preceding BMT.

The aim of this study was to address the question of whether BM donor-derived cells contribute to the endometrial SP population of recipients and, therefore, if they contribute to the endogenous source of SSC or not. To this end, a clinical model of female BMT recipients who received BM from human leukocyte antigen (HLA)-identical male donors was used.

## Materials and Methods

### Ethics Statement

Five female patients who received an allogeneic SC transplantation from male donors for the treatment of hematological malignancies at the Hematology Department, Hospital Universitario La Fe, Valencia, Spain from May to October in 2010, were enrolled in this study.

This project was approved by the institutional review board of Hospital Universitario La Fe registered under number 2009/0364, and all patients gave written informed consent.

### Patients and tissue samples

Recipients' ages ranged between 28 and 41 years. Transplant was performed to treat a variety of hematological malignancies: chronic myeloid leukemia (CML) in two patients; acute myeloblastic leukemia (AMbL) in two patients; and acute lymphoblastic leukemia (ALL) in one patient. All the patients received a busulfan-based myeloablative conditioning regimen without total body irradiation (TBI). Patients 1, 2 and 5 received transplantation from unrelated donor umbilical cord blood (UD-UCB). Patient 3 received an HLA-matched unrelated bone marrow transplant (UD-BMT). Finally, patient 4 was transplanted using mobilized peripheral blood stem cells (PBSC) from an HLA-matched sibling donor (see Table 1).

**Table 1:** Characteristics of transplant recipients.

Patient	Age	Duration of HRT (months)	Pregnancy after BMT	Time from transplantation to biopsy (months)	Indications for transplant	Hematopoietic Progenitors (age)
**1**	38	4	Yes	132	CML	CBSC (0)
**2**	29	15	No	30	AMbL	CBSC(0)
**3**	41	72	Yes	210	CML	BM (46)
**4**	34	36	No	116	ALL	PBSC (21)
5*	28	18	No	45	AMbL	CBSC(0)

CBSC, Cord Blood Stem Cell; BM, Bone Marrow; PBSC, Peripheral Blood Stem Cells; HRT, Hormone Replacement Therapy. (*atrophic endometrium).

Patients with secondary amenorrhea after transplantation received hormone replacement therapy (HRT) using a Gonadotropin-releasing hormone agonist depot (GnRH-a) (Decapeptyl 3.75, IPSEN, France) injected on day 21 of the previous cycle. Estradiol valerate (EV) (Progynova; Schering Spain, Madrid, Spain), 6 mgr daily, was initiated on day 2 of the cycle for at least 10 days, while progesterone (Utrogestan, Corne, Mexico), 800 mg per day, was added as previously described [41]–[42]. An endometrial tissue specimen was obtained with a Pipelle catheter (Gynetics Medical Products N.V, #4164 probet, Belgium) under sterile conditions from the uterine fundus at a minimum of 4 months from starting HRT. Histological sections from three patients without transplantation were used as positive controls to detect the absence of chromosome Y signals.

### Study design

The endometrial samples obtained from all five BMT recipients were divided and processed as formalin-fixed paraffin embedded and enzymatically disaggregated viable single cell suspensions. Paraffin embedded tissues were examined using fluorescent *in situ* hybridization (FISH) to target chromosomes X and Y, immunohistochemistry was done to immunophenotype endometrial XY cells, while a telomapping analysis was carried out for telomere quantification purposes. The cell suspensions from the same specimens were processed to isolate SP cells as previously described [20], [29] to be then FISH analyzed.

### Fluorescence In Situ Hybridization (FISH)

A fluorescence *in situ* hybridization analysis was performed with the specific probes for chromosomes X and Y (CEP Probes, Abbot). Paraffin embedded slides were deparaffinized at 60° overnight. Afterward, sections were cleared with xylene for 10 minutes, and twice re-hydrated in several washes with 100%, 85% and 70% ethanol for 5 minutes each. Then slides were rinsed with distilled water (dH_2_O) for 5 min at room temperature (RT). Antigen retrieval was done with citrate buffer 10 mM (pH 8.0) in a microwave within a humidified chamber for 5-min periods at a high temperature. For enzymatic digestion, hydrochloric acid 0.2 M was used for 10 minutes, and slides were continuously treated with Proteinase K 10 µg/mL for 10 minutes in a bath at 37°C. Slides were rinsed with saline-sodium citrate buffer (2× SSC) 5 min and fixed with 1% paraformaldehyde buffer for 10 minutes before being rinsed again with 2× SSC buffer 5 min and air dried.

Next, slides were dehydrated in a battery with ethanol prior to hybridization with the probes. The mixture of probes was added to the slides, covered with coverslips and incubated in a humidified chamber at 80°C for 5 minutes for denaturation and at 37°C for 72 hours for hybridization. Afterward, slides were washed with 0.4×SSC buffer / 0.3% NP40 (nonyl phenoxypolyethoxylethanol) pH 7.5 in a bath at 80°C for 2 minutes and then with 2×SSC/0.1% NP40 pH 7.2 at RT. Finally, nuclei were stained with diamidino-2-phenylindole dihydrochloride (DAPI).

Tissue controls for the X and Y signals were the endometria from patients without transplantation and fetal testes, respectively.

### Quantification of XY donor-derived cells

Slides were observed under a fluorescence microscope (Leica DM 6000 B/M) at a 63× magnification with oil immersion objective. Female cells, represented by two X chromosomes, shared the same color (spectrum orange) FISH signals, corresponding to the X chromosomes, whereas male cells, XY chromosomes, displayed two different colors (spectrums orange and green) FISH signals, representing both chromosomes. A range of 1000–1500 nonoverlapping cells was counted to analyze the percentage of XY cells within the recipient endometrium. Total numbers of XX and XY cells were quantified with the Image-Pro Plus Software Version 6.3 (MediaCybernetics).

### Confocal telomere quantitative fluorescence in situ hybridization

The telomapping technique [43]–[44] was followed to localize the putative niches of SSC represented by the hot regions associated with large telomeres.

The above-described telomapping method was based on the telomere length quantification on interphase nuclei. Therefore, slides were hybridized with a PNA-tel Cy3-labeled probe. Briefly, DAPI and Cy3 signals were acquired simultaneously in separate channels using a confocal ultra spectral microscope (Leica TCS-SP2-A-OBS-UV). The binary DAPI mask was applied to the matching Cy3 to obtain a combined image with each nucleus telomere fluorescence information. Cy3 fluorescence intensity (telomere fluorescence) was measured as the “average gray value” (total gray value/nuclei area) units (arbitrary units of fluorescence). These “average telomere fluorescence” values always represent the average Cy3 pixel intensity for the total nuclear area, and not the average value of individual telomere spot intensities, therefore ruling out that differences in nuclear size may influence telomere length measurements.

### Immunofluorescence and confocal analysis for CD45, CD9 and Vimentin

To immunophenotype the XY donor-derived cells, CD45, CD9 and Vimentin were analyzed. After hybridization with sex chromosomes, slides were washed with phosphate buffered saline (PBS). Prior to antibody incubation, slides were blocked with PBS supplemented with 3% Bovine Serum Albumin (BSA), 5% Normal Goat Serum (NGS) and 0.05% Tween-20 for one hour at RT to treat nonspecific unions. Following this, overnight incubation with a primary antibody was applied; the specific antibodies used in this study were mouse anti-human CD45 (BD Pharmingen, 555480), mouse anti-human CD9 (Abcam, 49325) and mouse anti-human Vimentin (Vm) (Abcam, 8069). Next, slides were washed with PBS and incubated with the appropriate fluorescent secondary antibody: Alexa 633 F(ab′)2 fragment goat anti-mouse (Invitrogen, A-21053). Finally, slides were rinsed with PBS and dH_2_O. Furthermore, nuclei were stained with DAPI.

The controls used were spleen for the CD45 expression endometrium containing epithelial glands and stroma for CD9 and Vm, respectively.

Double labeling by FISH and immunofluorescence against CD45-CD9-Vm were observed by confocal microscopy. Images were acquired with a Leica TCS SP2 AOBS (Leica Microsystems Heidelberg GmbH, Mannheim, Germany) inverted laser scanning confocal microscope using a 63× Plan-Apochromat-Lambda Blue 1.4 N.A. oil immersion objective. The excitation wavelengths for fluorochromes were 488 nm for FITC (green), 561 nm for TRITC (orange/red), 633 nm for Alexa633 (far red) and 405 nm for DAPI (blue). Two-dimensional pseudo color images (255 color levels) were acquired with a size of 1024×1024 pixels. All the confocal images with the same settings and fluorescence distribution were analyzed using the Leica “Leica Lite” Confocal Software, version 2.61.

The combination of different fluorophores allowed the characterization of the XY cells with their corresponding phenotype.

### Isolation of endometrial side population cells

Epithelial and stromal cell suspensions from an endometrial biopsy were obtained by following a well-established protocol with minor modifications [44]. Samples were washed to discard remnants of blood and mucus to be then minced into small fragments which were enzymatically digested with collagenase type IA (Sigma-Aldrich, Spain) at 10 mg/ml in Dulbecco's Modified Eagle Medium (DMEM) (Sigma-Aldrich, Spain). Gravity sedimentation and membrane filtration allowed the separation of stromal cells and epithelial glands on a size basis [45]. Cell suspensions were treated with erythrocyte lysis solution and cell viability was also assessed with Propidium Iodide (PI; 5 µg/ml (Sigma-Aldrich, Spain)) to exclude any dead cells to be analyzed.

Single cell suspensions were incubated in a bath at 37°C for 90 minutes with Hoechst 33342 dye 10 µg/mL (Sigma- Aldrich, Spain) to identify and isolate side population (SP) cells as putative endometrial SSC and non side population cells (NSP) [20].

After incubation, cells were centrifuged and resuspended in Hank's Buffered Salt Solution buffer (HBSS) (Gibco, Invitrogen, Spain) supplemented with 2% Fetal Bovine Serum (FBS) (Gibco, Invitrogen, Spain) and HEPES 10 mM pH 7.2 (Sigma- Aldrich, Spain). Those cells labeled with Hoechst were analyzed and sorted by a MoFlo® (Dako, Denmark, http://www.dako.com) jet-in-air high speed sorter. Excitation was performed with a water-cooled Enterprise II ion laser (Coherent, CA, USA), which operated at the 351 nm and 488 nm wavelengths, and worked at 30 mW. Hoechst 33342 blue and red fluorescences were detected through 405/30 and 670/20 nm band-pass filters, respectively, by measuring signals on a linear scale. PI fluorescence was detected through a band-pass filter of 613/20 on a logarithmic scale. The gates for cell sorting were defined to collect live cells with low Hoechst 33342 fluorescence (SP fraction), as well as live cells with high Hoechst 33342 fluorescence (NSP fraction).

### FISH in cell suspensions

SP and NSP cells were cultured under hypoxic conditions (1–2% O_2_) for 3 weeks. Cells were trypsinized, treated with chloride potassium 0.075 M and fixed with 3 methanol∶1 acetic acid to proceed with the FISH analyses.

Slides with fixed cells were dehydrated in a battery with ethanol (70%, 85% and 100%) for one minute each, and were air-dried. A mixture of centromeric probes for chromosomes X (spectrum orange), Y (spectrum green) and 18 (spectrum aqua) (Poseidon probes, Kreatech, Spain) was added to the slides, which were covered with coverslips and incubated in a humidified chamber at 73°C for 5 minutes for denaturation and at 37°C for overnight hybridization. Afterward, slides were washed firstly with 0.4×SSC/0.3% NP40 pH 7.5 in a bath at 73°C for 2 minutes and secondly with 2×SSC/0.1% NP40 pH 7.2 at RT. Finally, nuclei were stained with DAPI.

## Results

### XY cells of donor origin in the endometria of BMT recipients

The BMT recipients' epidemiological characteristics in terms of age, HRT duration, if they have been pregnant or not after BMT, the time from BMT to the endometrial biopsy, indications for transplant, and type of hematopoietic precursors used, are all shown in Table 1.

**Figure 1:**
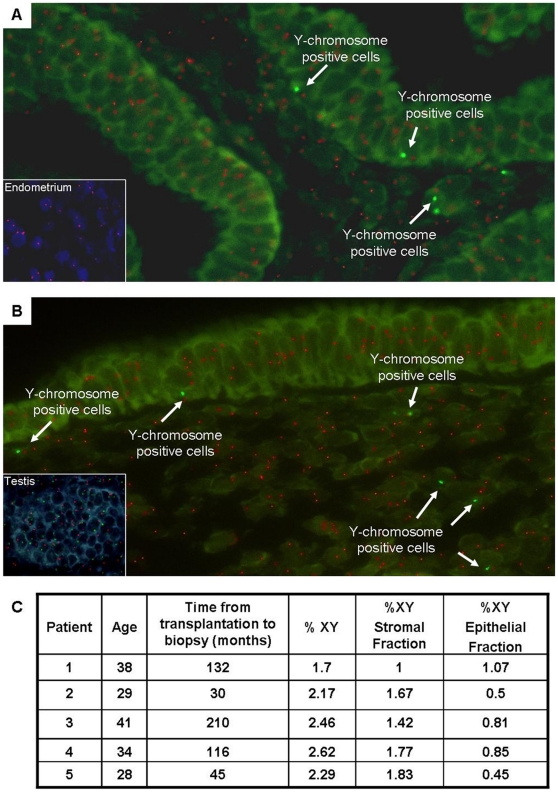
Detection and quantification of XY cells from donor origin in the endometria of BMT recipients. (A&B) Human endometrial sections from 2 different patients, who underwent BMT, showing a positive FISH signal corresponding to chromosome Y (green signal) and chromosome X (orange signal) along both the epithelial and stromal compartments. Presence of donor-derived cells (chromosome Y-positive cells) in the endometrium is indicated by arrows. Lower panels containing control slides: (A) endometrium as a positive control for the chromosome X signal and (B) fetal testes as a positive control for the chromosome XY signal. (C) Table summarizing the characteristics of the recipients, the percentages of XY-positive cells obtained in the whole endometrium, plus the epithelial and stromal fractions. All the images were acquired using a 63×/1.6× oil immersion objective.

We examined the presence of donor-derived cells (chromosome Y-positive cells) in BM transplant recipients' endometria using FISH analyses. Tissue sections were analyzed by two observers with a total of 5 visual fields per sample scored (around 1000–1500 cells). The results reveal the existence of XY donor-derived cells in all the endometrial sections from the recipients analyzed (Figure 1A and 1B). The donor-derived cells corresponding to chromosome Y-positive cells accounted for 1.7–2.62% of the total cell count, with less contribution to the epithelial compartment (ranging from 0.45% to 0.85%), while the percentage of chromosome Y-positive cells in the stromal compartment oscillated from 1.0% to 1.83%. It is interesting to note that no correlation was found between the time from transplantation to biopsy (from 30 to 210 months) and the percentage of the chromosome Y-positive cells found in the endometrium (Figure 1C).

Control FISH analyses included the endometria of healthy patients without transplantation who had a previous pregnancy carrying a boy to avoid the possibility of contamination of chromosome Y-positive cells via materno-fetal chimerism and male tissues from fetal testes. As expected, all the cells in our controls exhibited orange (chromosome X-positive cells) signals (Figure 1A, lower panel), whereas the ratio of the chromosome Y- to chromosome X- positive cells was confirmed in male testes (Figure 1B, lower panel). Moreover, the possibility of cell fusion was ruled out given the absence of aneuploidies.

### Immunophenotyping of the XY donor-derived cells in the endometria of transplanted patients

In order to clarify the final differentiation of the XY donor-derived cells in the endometrium, we used a battery of markers composed of CD45 for cells of a hematopoietic origin, CD9 and Vm for endometrial epithelial and stromal cells, respectively. These markers were co-localized in combination with chromosome Y detection.

**Figure 2:**
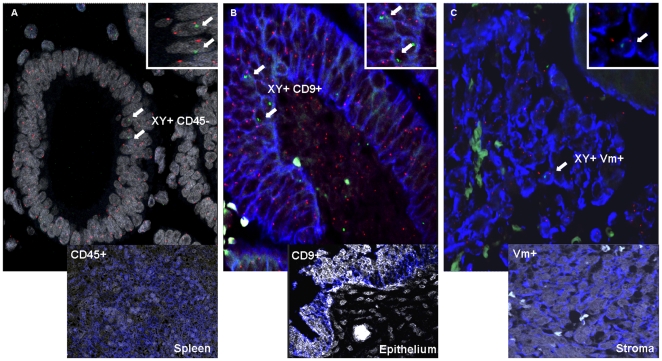
Immunophenotyping of the XY donor-derived cells in the endometria of transplanted patients. (A) XY donor-derived cells (indicated by arrows, orange and green signals) in the absence of the hematopoietic marker CD45+ (blue signal) in the recipients' endometria. Lower panel: spleen as a positive control for CD45 (blue). (B) Co-localization of XY donor-derived cells (indicated by arrows) with epithelial marker CD9 (blue) in recipients' endometria indicate the coexistence of XY+CD9+ cells in epithelial glands. Lower panel: epithelial endometrium as a positive control for CD9 (blue). (C) Co-localization of XY donor-derived cells (indicated by arrows) with stromal marker Vm (blue) in recipients' endometria, indicated by the overlapping of the XY+Vm+ expression in the stromal fraction. Lower panel: stromal endometrium as a positive control for Vm (blue). All the images were acquired using a 63×/1.7× oil immersion objective with a confocal microscope. Note that DAPI signals are shown in gray in all the images.

As a result, we observed that none of the XY donor-derived cells were CD45-positive, which rules out the possibility of hematopoietic progenitors and contamination by resident leukocytes originating from BMT (Figure 2A). All the XY donor-derived cells present in the glands were CD9-positive (Figure 2B), and all the XY donor-derived cells in the stroma colocalized with Vm marker (Figure 2C), suggesting a final tissue-specific differentiation of XY donor-derived cells. Finally, spleen and endometrium were used as positive controls to assess the specific immunoreactivity of the antibodies used (Figure 2).

### Analysis of the telomere length regions in the endometria of BMT recipients

**Figure 3:**
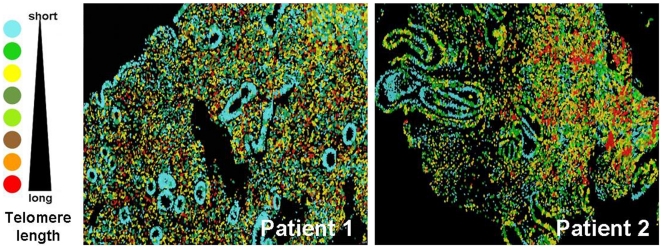
Analysis of the telomere length regions in the endometria of BMT recipients. Assessment of telomere length in the endometrial sections from BMT recipients: note that patients 1 and 2 are represented. A color code on the left-hand side of the image symbolizes the degree of colors corresponding to telomeric length, where long and short telomeres are represented in red and blue, respectively. Also note how the regions associated with long telomeres are located mainly in the stromal compartment.

In order to search for the localization of possible niches of endometrial SSC within the endometrium and whether XY donor-derived cells are part of them, we examined the telomere length in the endometrial sections from both BMT recipients and nontransplanted patients by telomapping [43]–[44]. In all the endometrial sections, hot regions associated with long telomeres were located specifically in the stromal compartment. We did not find differences in terms of telomere length regions with or without BMT. In BMT recipients, most XY donor-derived cells were randomly distributed and could not be associated with long telomere regions (Figure 3).

### Isolation of endometrial SP cells and FISH analyses of BMT recipients

**Figure 4:**
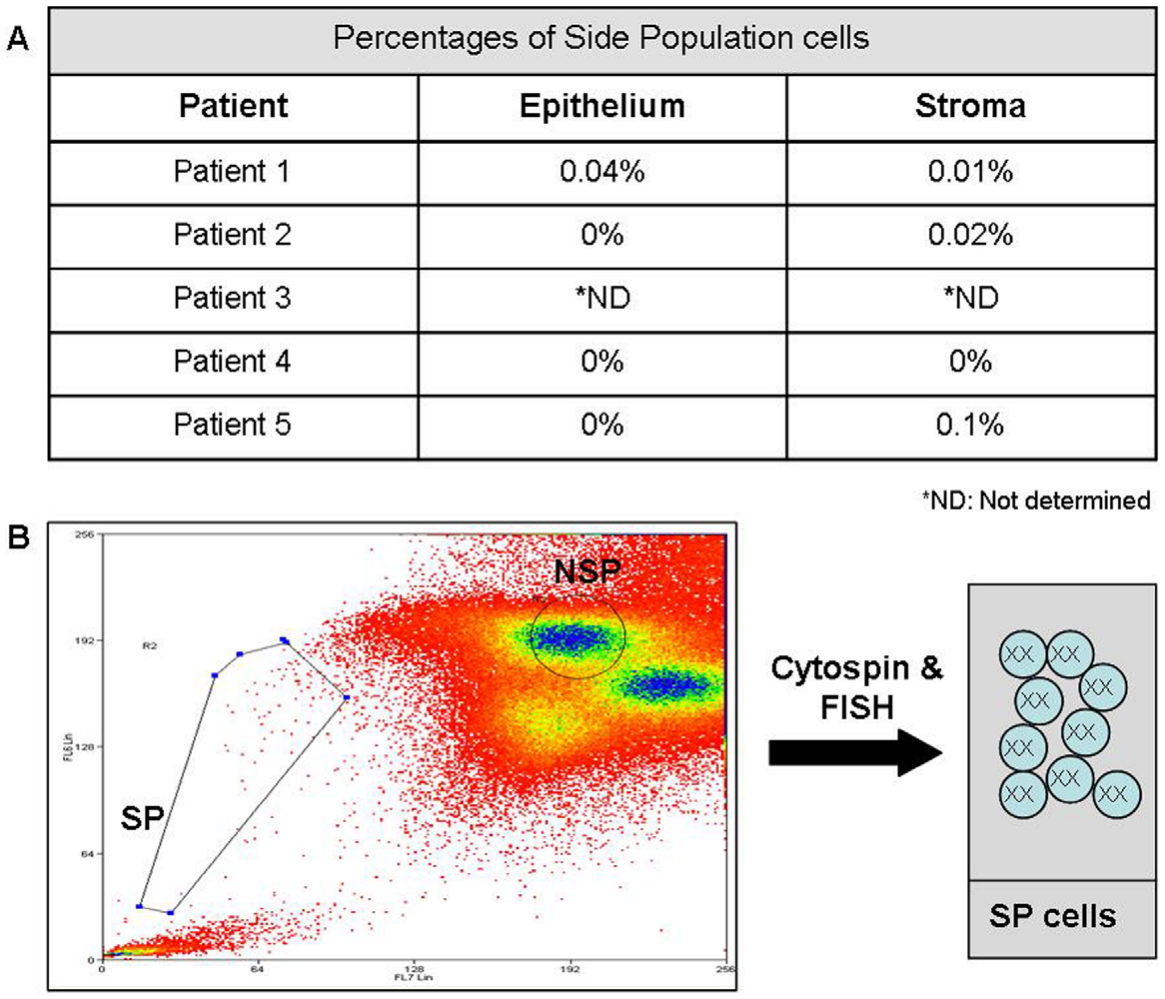
Isolation of endometrial SP cells and FISH analyses from BMT recipients. (A) Table indicating the percentages of freshly endometrial SP cells obtained in all 5 patients by a cell sorter analysis. Percentages are provided for both the epithelial and stromal fractions. (B) Diagram showing a typical SP graph and the results of the FISH analyses of epithelial and stromal SP cells, revealing the absence of XY donor-derived cells.

In order to investigate to what extent the donor-derived cells from male donors participate in endometrial SP, which has been demonstrated to constitute the SSC population [18], [20], [29], we first isolated this subset of cells from the epithelial and stromal fractions in all five BMT recipients. One interesting finding was that the percentage of freshly epithelial SP cells was 0% in four patients, and that 1 SP represented 0.04% of the total cell population in only patient. In patients 1, 2 and 5, freshly stromal SP percentages ranged from 0.01% to 0.1% (Figure 4A), and SP cells were not isolated in patients 3 and 4 (Figure 4A). Unexpectedly, the viability of all the analyzed samples was very low compared to nontransplanted patients [20].

The FISH analyses of the epithelial and stromal SP from transplanted patients revealed the absence of XY donor-derived cells (Figure 4B). The number of cells cultured during 3 weeks in each sample is strictly related to the initial number of SP cells obtained from the biopsy (mean: SP 400 cells/NSP 100.000 cells). In it context, FISH analysis was only performed in cultured cells. It is noteworthy that one XY cell was observed in a total of 100 FISH analyzed cells in the stroma NSP fraction of patient 4.

## Discussion

We herein confirm with a pathological model of females who received BMT from HLA-identical male donors to treat hematologic cancers that the XY cells of a BM donor origin migrate to the recipient's endometrium, and transdifferentiate and contribute to the cell composition of the stroma and the epithelial compartment. We demonstrate that the presence of XY donor-derived cells in the recipient's endometrium ranges from 1.7% to 2.62%. We identify and localize 0.45% to 0.85% of the XY donor-derived cells in the epithelial compartment displaying CD9 marker, and 1.0% to 1.83% of the XY donor-derived Vm-positive cells in the stromal compartment. This scenario supports the capacity of BM donor cells to give rise to endometrial tissue.

Moreover, different studies have demonstrated that multipotent human BM donor-derived cells differentiate into cell types of the functionally competent endometrium. For the first time, Taylor identified in 2004 donor cells in endometrium recipients after BMT, and confirmed their typical structural epithelial and stromal features and their corresponding hematopoietic CD45 negative phenotype [37]. In line with this, Ikoma [40] assessed the unexpected plasticity of BM cells giving rise to a functional endometrium. This they demonstrated first by the typical expression pattern in the epithelial (Cytokeratin, Estrogen Receptor-α) and stromal (CD10) compartments in the absence of CD45 marker, and second by successful pregnancies in two of the three patients analyzed. The presence of donor-derived endothelial cells [39] in the endometrium has also been described to contribute to the formation of new blood vessels (neovascularization) in this tissue in ongoing pregnancy (one case).

Similar endometrial engraftment has been demonstrated In two murine models, Du and Taylor demonstrated the *de novo* development of the endometrium after injury due to BM stem cells, suggesting an implication between endometriosis and immune disorders [38]. Following BMT, Bratincsak and cols detected donor-derived cells in the epithelial and stromal endometria of recipient animals, where CD45 positive cells play an important role in stromal compartment and were found to be a putative source of cells for epithelium regeneration [46].

Furthermore, our study contributes new evidence demonstrating that XY donor-derived cells can be considered a limited exogenous source of transdifferentiated endometrial cells rather than a cyclic source of bone marrow-derived stem cells. First, they were not incorporated into the endometrial SP by considering, at least in part, the SSC population because two independent groups have demonstrated SP ability to regenerate the human endometrium after cell suspension injection into the dermis [20] or the renal capsule [18], [29] of NOD-SCID mice. Nonetheless, the possibility of BM-derived cells inducing or favoring the endometrial SP population in response to a physiological demand cannot be ruled out. Secondly, XY donor-derived cells were not associated with the SC niches assessed by telomapping as long telomere regions [43]–[44]. This approach enables the analysis of those cells of different telomeric lengths, as recently used to identify somatic stem cells in adult tissues and organs like skin [43] and human colon [47].

Finally, engraftment percentages were very low and no correlation was found between the time from transplant and engraftment efficiency, suggesting random terminal differentiation, which may even be due to hormonal regulation, as demonstrated by the absence of CD45.

In physiological terms, the transient infiltration of BM-derived cells occurs in each menstrual cycle [11], [37]. Our results indicate that a final differentiation from the BM cell population to endometrial tissue might occur, but that this event is not related with endogenous endometrial stem cell capability since we have demonstrated that no endometrial SP was of a BM origin. Therefore, we suggest that BM cells do not contribute to the endometrial SC pool, but might contribute to form differentiated endometrial cells, be it to a limited extent under pathologic conditions. Similarly, XY donor-derived cells were not detected in the recipients' endometrial SP fraction. We considered that in the case of non-stem cells proliferate predominantly in vitro we must be able to detect any XY cells as non dividing cells. Our previous publication showed the capability of endometrial SP lines to regenerate the whole endometrium in an animal model demonstrating the presence and maintenance of endometrial SP cells with stem cell features under culture conditions [29]. It seems most unlikely that chromosome Y might be lost during engraftment, as reported in the lung of a murine transplantation model [48], because endometrial cells do not form the heterokaryon, and cell fusion has been ruled out by chromosomal analyses.

Hypothetically, activation of dormant BM stem cells after stress or injury could implicate the mobilization of these cells from BM [49]. The questions that arise in connection with our data are about the mechanisms controlling the differentiation of BM cells into different endometrial cell types, and why BM contribution is more important for the stromal than it is for the epithelial compartment. Therefore, could BM stem cells provide factors that favor the reactivation of the damaged endometrial niche?

It is surprising that in our study we discovered that SP reduced if compared to nontransplanted patients because of chemotherapeutic agents. Nevertheless, the largest retrospective study, which consists in 229 centers of the European Group for Blood and Marrow Transplantation (EBMT) and includes patients who conceived naturally or by assisted reproductive techniques (ART) after BMT, demonstrates that the recipients' endometria were functional and concludes that the outcome of a pregnancy after BMT is likely to be successful [50].

The latest publication about this topic assessed endometrial renewal in an Asherman's syndrome patient throughout the intrauterine administration of BM stem cells. The clinical evidence deriving from this study confirms the feasible stimulation of dormant endometrial stem cells to a cell proliferation status to help regenerate the damaged endometrium [51].

In short, we confirm the presence of XY donor-derived cells of a BM origin in the endometria of transplanted recipients, which suggests a high plasticity process to renew endometrial tissue. Furthermore, we demonstrate that XY donor-derived cells from BM can be considered an exogenous source of transdifferentiated endometrial cells, be it to a limited extent, rather than a cyclic source of BM-derived stem cells.
